# Nutritional Variation Among Foods Included in an Ecuadorian Food Composition Database Using Multivariate Analysis

**DOI:** 10.3390/nu18172910

**Published:** 2026-09-04

**Authors:** Orlando Meneses Quelal

**Affiliations:** Facultad de Industrias Agropecuarias y Ciencias Ambientales, Carrera de Alimentos, Universidad Politécnica Estatal del Carchi (UPEC), Tulcán 040101, Ecuador; orlando.meneses@upec.edu.ec

**Keywords:** food composition database, nutrient profiling, multivariate analysis, missing data, principal component analysis, hierarchical clustering

## Abstract

**Background/Objectives:** This study characterized compositional variation among 996 foods and beverages retained from the Universidad San Francisco de Quito Food Chemical Composition Table after record-level quality control. **Methods:** Nineteen nutritional variables were evaluated using descriptive statistics, heteroscedastic one-way analysis of variance, rank correlations, principal component analysis, and hierarchical clustering. Missingness was substantial and non-random across food groups; 427 records contained all 19 variables and were used for multivariate analyses. **Results:** Welch tests with Holm correction identified food-group differences for all variables, although the magnitude of group separation ranged from small for vitamin A (η^2^ = 0.029, 95% CI 0.016–0.061) to large for carbohydrates (η^2^ = 0.590, 95% CI 0.555–0.632) and energy (η^2^ = 0.588, 95% CI 0.530–0.654). Spearman analysis showed strong expected compositional associations between total fat and monounsaturated fatty acids (ρ = 0.956) and between total fat and saturated fatty acids (ρ = 0.937), together with non-arithmetic associations involving protein, phosphorus, zinc, cholesterol, and vitamin B12. Parallel analysis retained four principal components, which explained 61.4% of total variance; the first two accounted for 40.2% and primarily represented energy-lipid-mineral covariation and lipid composition. Ward clustering in the full 19-dimensional standardized space supported a three-cluster solution (silhouette = 0.482; median subsampling adjusted Rand index = 0.845), comprising a broad mixed profile, a fiber- and mineral-dense profile, and an oil-dominant profile. **Conclusions:** These results describe compositional patterns within the USFQ database and provide a reproducible basis for database harmonization, nutrient profiling, and hypothesis generation. They do not estimate dietary intake, food availability, nutritional adequacy, or health outcomes in the Ecuadorian population.

## 1. Introduction

Behind every dietary recommendation, every food consumption survey, and every nutrition label lies a piece of data that is rarely questioned: the energy and nutrient content attributed to each food. That figure almost always comes from a food composition table, a systematic inventory that translates the enormous variety of available foods into comparable numbers. Without reliable, up-to-date, and representative composition tables, nutritional research loses much of its capacity to estimate intakes, detect deficiencies, and evaluate policies [1,2].

Building these tables is no trivial task. It requires compiling laboratory chemical analyses, values taken from international tables, recipe-based calculations, and, increasingly, information declared on packaged-product labels, all under identification and unit criteria that must remain consistent across sources [3]. For this reason, organizations such as FAO, INFOODS, and its regional network LATINFOODS have promoted methodological harmonization of composition tables in Latin America since 1984, so that data from one country can be compared with those of another [4]. Later initiatives, such as the standardization of the databases used in the Latin American Study of Nutrition and Health (ELANS), have shown that, even within the region, differences between national tables can introduce considerable systematic errors if they are not harmonized before comparing countries [5].

Food composition databases are intrinsically multivariate because energy, macronutrients, fatty acid fractions, minerals, and vitamins covary within foods. Analyses based only on isolated means can therefore obscure compositional gradients and combinations that are relevant to food classification. A review of statistical applications in food composition databases found that clustering, ordination, and association analyses have been used across national and regional datasets, although methodological reporting and treatment of missing values remain heterogeneous [6,7]. In South Africa, principal component analysis of 971 foods and 28 nutrients identified eight patterns explaining 73.4% of variance and showed that data-derived structures can align with food-based dietary guidance [8]. Comparable applications demonstrate that multivariate analysis is useful for describing internal database structure, but they also show that conclusions must remain bounded by the provenance, completeness, and representativeness of the underlying records. More recently, artificial intelligence and machine-learning approaches have opened additional possibilities for analyzing large volumes of nutritional data, although their specific application to food composition databases remains incipient [9].

Ecuadorian food composition information has developed through several complementary initiatives. The Cuenca Food Composition Table compiled 129 traditional foods and provided an initial locally oriented reference [10]. A subsequent review documented that nutrient-intake estimation in Ecuador has often relied on heterogeneous national and foreign tables, creating risks of non-equivalent food matching and systematic measurement error [11]. The USFQ Food Chemical Composition Table, published in the Bitácora series, expanded the number of listed foods and the range of reported nutrients [12]. Its value lies in the breadth of the compilation, but its records derive from multiple sources and should not be interpreted as a probabilistic sample of foods available, purchased, or consumed in Ecuador. Locally relevant nutritional evidence in Ecuador also includes reviews of dietary intake and nutritional status among children [13] and analyses of ancestral Andean foods such as quinoa and amaranth that remain compositionally under-characterized [14].

The database includes fresh foods, culinary ingredients, prepared foods, beverages, and packaged products. This heterogeneity permits comparison of diverse compositional profiles, but it does not provide the ingredient and processing information required to assign NOVA categories systematically [15,16]. Consequently, packaged products were analyzed according to their recorded food groups and nutrient values without being classified as ultra-processed. This distinction is necessary because compositional data alone cannot establish processing category, consumption frequency, dietary exposure, or population-level contribution.

The objective of this study was to characterize nutritional variation among foods included in the USFQ Food Chemical Composition Table using descriptive, inferential, and multivariate methods. The analysis quantified the proportion of variability associated with food-group classification, evaluated robust pairwise nutrient associations, identified latent compositional dimensions, and classified complete records into empirically supported nutritional profiles. A further objective of the revision was to quantify missingness and evaluate how complete-case selection affected the representation of food groups. The intended contribution is methodological and compositional: to provide a transparent analysis of this database that can support future harmonization, food-level nutrient profiling, and the formulation of testable hypotheses for studies that incorporate consumption or market data.

## 2. Materials and Methods

### 2.1. Origin and Provenance of the Database

This secondary analysis used the Food Chemical Composition Table Based on Nutrients of Interest for the Ecuadorian Population, published by Universidad San Francisco de Quito in December 2021 as Bitácora volume 11 [12]. The source document compiled values from the following nine origins: Central American, Peruvian, Colombian, Bolivian, and Cuenca—Ecuador food composition tables; USDA FoodData Central; nutrition labels; scientific articles; and standardized recipe calculations based on EuroFIR procedures [10,17,18,19,20,21,22]. The database is therefore a heterogeneous compilation and not a survey of food availability, purchasing, or consumption.

The PDF was reconstructed using coordinate-aware table extraction. Word objects were read with their page and horizontal and vertical coordinates, grouped into rows according to vertical alignment, and assigned to fixed nutrient columns according to the header-defined horizontal intervals. Multiline food names were concatenated before numeric fields were parsed. Decimal symbols and units were standardized, while blank cells were retained as unavailable and were never converted to zero. Each record preserved the source page, food group, reporting basis, source code, Spanish and English food names, and the 19 nutrient fields. The reconstructed file contains a page-level trace for every record, permitting direct return to the source table during auditing.

### 2.2. Verification and Quality Assurance of the Records

A preliminary non-coordinate extraction was retained only as a diagnostic comparison. It contained 202 records with probable column displacement and 296 macronutrient values outside physiologically plausible ranges among 890 evaluable records, including negative values and protein, fat, or carbohydrate concentrations above 100 g/100 g. Because those errors were systematic, the preliminary file was discarded and no result in this study was derived from it. All analyses were performed on the coordinate-reconstructed database.

Quality control was implemented at record and cell levels. All nutrient values were required to be numeric and non-negative. Energy was screened within 0–900 kcal/100 g or 100 mL; protein, total fat, carbohydrates, fiber, and individual fatty acid fractions within 0–100 g/100 g or 100 mL; and micronutrients within non-negative domains. Reported energy was compared with the Atwater estimate calculated from protein, fat, and carbohydrates as a screening diagnostic rather than an exclusion rule because fiber, polyols, organic acids, alcohol, and rounding can create legitimate discrepancies. Of 1002 reconstructed records, 996 passed the automated rules. Six records were excluded because they contained negative macronutrients, values exceeding the physical reporting basis, or unresolved Atwater discrepancies coupled with source-table ambiguity. A second robust discontinuity screen applied to log-transformed micronutrient distributions identified six isolated cells, iron values of 392 and 130 mg, zinc values of 272, 213, and 153 mg, and a vitamin B12 value of 357 µg. These values were separated sharply from the next observations and were inconsistent with adjacent row structure; they were recoded as unavailable without excluding the corresponding foods. The complete audit trail is supplied with the reconstructed dataset.

After six cell-level recordings, mean completeness was 71.4%. Completeness ranged from 46.6% for folate and 48.6% for vitamin B12 to 99.9% for energy. A total of 427 foods, 42.9% of the 996 retained records, contained all 19 variables. Complete-case inclusion differed substantially by food group, with inclusion rates of 82.1% for vegetables, 74.0% for meat and processed meats, 70.5% for fish and seafood, 15.7% for sugars, 1.0% for snacks, and 0% for beverages and liquids. Principal component and cluster analyses were therefore restricted to 427 records, and their results were interpreted as patterns of the complete-case subset rather than of the full database. This level of completeness is comparable to other food composition databases, where missing data are a widespread problem more often addressed through exclusion of incomplete cases than through statistical imputation, as was chosen here [23,24]. Descriptive, group-comparison, and pairwise-correlation analyses used all observations available for each variable.

### 2.3. Categorization of Foods by Group

The following original eleven-group classification was retained: cereals, plantains, and tubers; fruits; vegetables; legumes; meat and processed meats; fish and seafood; milk and dairy products; fats and nuts; sugars; snacks; and beverages and liquids. Group labels were treated as database categories and not as indicators of consumption or processing level. For each nutrient analysis, the number of observations contributed by every group was recorded explicitly.

### 2.4. Nutritional Variables Considered

Nineteen variables were selected because they constitute the common analytical scope of the USFQ table and represent complementary dimensions of nutritional composition. They comprised energy; protein, total fat, carbohydrates, and dietary fiber; saturated fatty acids (SFAs), monounsaturated fatty acids (MUFAs), and polyunsaturated fatty acids (PUFAs); cholesterol; calcium, phosphorus, iron, potassium, sodium, and zinc; and vitamins C, A, folate, and B12. Selection was therefore based jointly on availability in the source table, coverage of macronutrient and micronutrient domains, relevance to nutrient-density assessment, and public-health relevance to deficiencies and cardiometabolic risk [25,26,27,28,29,30]. Vitamin A was standardized as micrograms of retinol activity equivalents, µg RAE, and all values were expressed per 100 g of edible portion or per 100 mL for beverages and liquids.

### 2.5. Statistical Processing of the Data

Descriptive statistics were calculated for each nutrient using all available observations and included the mean, standard deviation, median, interquartile range, minimum, maximum, and sample size. Distributional diagnostics comprised skewness, group-wise Shapiro–Wilk tests where sample size permitted, and the Brown–Forsythe test based on absolute deviations from group medians. Strong skewness, unequal variances, unequal group sizes, and nutrient-specific missingness were observed. Consequently, between-group inference was based on Welch one-way analysis of variance rather than the equal-variance F test. Groups with fewer than two observations or zero within-group variance for a nutrient were excluded from that nutrient-specific Welch model and the resulting degrees of freedom were reported. Nineteen primary Welch tests were adjusted using the Holm family-wise error procedure. Kruskal–Wallis tests with Holm adjustment were used as a distribution-free sensitivity analysis.

Group separation was summarized using η^2^ and the less biased ω^2^ calculated from one-way sums of squares [31,32]. Uncertainty for η^2^ was estimated with 1200 stratified bootstrap resamples that preserved the observed sample size of each food group. Nutrient associations were estimated primarily with Spearman rank correlations because of skewness and influential observations. The 171 pairwise significance tests were adjusted using the Holm procedure. Pearson correlations were retained as a sensitivity analysis, and partial rank correlations were calculated after residualizing ranked nutrient values for food-group indicators to assess the extent to which associations persisted within the database classification. Correlations involving total fat and its component fatty acids, or energy and energy-yielding macronutrients, were interpreted as expected compositional or arithmetic dependencies rather than novel biological discoveries.

Principal component analysis was performed on the 427 complete records after Z standardization of all 19 variables [33]. Reported loadings are correlations between each standardized nutrient and the component score, calculated as the eigenvector coefficient multiplied by the square root of the corresponding eigenvalue. Component retention was assessed using eigenvalues, cumulative variance, and Horn parallel analysis with 1000 independently permuted reference matrices; a component was retained when its observed eigenvalue exceeded the 95th percentile of the corresponding reference eigenvalue. Hierarchical agglomerative clustering used Euclidean distance and Ward linkage in the complete 19-dimensional standardized space [34,35]. Solutions from two to ten clusters were compared using the silhouette coefficient, Calinski–Harabasz index, Davies–Bouldin index, minimum cluster size, and stability under 100 random 80% subsamples quantified by the adjusted Rand index. The PCA projection was used only for visualization and not for cluster validation. Analyses were conducted in Python 3 using NumPy 2.4.1, SciPy 1.17.0, scikit-learn 1.8.0, statsmodels 0.14.6, and Matplotlib 3.10.8. The reconstructed dataset, audit record, analysis outputs, and executable code are provided as Supplementary Files.

## 3. Results

### 3.1. Overview of the Analyzed Food Set

The quality-controlled analytical dataset comprised 996 records from the USFQ table distributed across eleven food groups (Table 1). Cereals, plantains, and tubers contributed 178 records, followed by meat and processed meats with 123, fruits with 111, and beverages and liquids with 111. These frequencies describe the composition of the database and should not be interpreted as market availability or dietary consumption.

Data provenance remained heterogeneous: 37.3% of records originated from packaged-product nutrition labels, 34.7% from the Central American table [17], 19.9% from USDA FoodData Central [21], and 3.7% from the Cuenca–Ecuador table [10]. Complete-case selection was strongly associated with food group, χ^2^(10) = 307.58, *p* < 0.0001, and Cramér’s V = 0.556. The 427 complete records overrepresented vegetables, meat, fish, and seafood and excluded all beverages and all but one snack. Compared with records lacking at least one nutrient, complete cases had higher protein and lower carbohydrate and MUFA distributions, with rank-biserial coefficients of 0.306, −0.268, and −0.338, respectively. These differences demonstrate that the PCA and clustering subset was compositionally selective.

### 3.2. Nutritional Profile by Food Category

Food-group summaries showed substantial heterogeneity, but reliability depended on nutrient-specific sample size (Table 2). Fats and nuts had the highest mean energy and total-fat concentrations, whereas vegetables had the lowest mean energy and fat. The beverage and liquid group combined low-energy drinks with a small number of highly concentrated products, producing a large standard deviation. Calcium for beverages was available for only one record and zinc for none; these estimates were therefore not interpreted.

Meat and processed meats and fish and seafood had the highest mean protein concentrations, while legumes represented the most protein-dense plant group. The magnitude of these differences is compositional rather than dietary because serving size, frequency of consumption, digestibility, and bioavailability were not included. Sample sizes shown in Table 2 also indicate that mineral comparisons were based on fewer observations than macronutrient comparisons in several groups.

Total fat was concentrated in fats and nuts, snacks, and milk and dairy products, while carbohydrates predominated in sugars and cereals, plantains, and tubers. These contrasts reflect the database categories and the underlying ingredients used to define them. They also explain part of the strong food-group effects observed in the robust omnibus analyses.

Legumes had the highest mean dietary-fiber concentration and dairy products had the highest mean calcium concentration. Interpretation of extreme means required attention to within-group dispersion and sample size. For example, the calcium mean for fats and nuts was influenced by calcium-rich seeds, while estimates for snacks and beverages were based on six and one observations, respectively. The revised analysis therefore emphasizes uncertainty and coverage rather than treating every group mean as equally precise.

### 3.3. Contribution of Food Group to Nutritional Variability

Brown–Forsythe tests indicated heterogeneity of variance for all nutrients, and most group distributions departed from normality. Welch analysis was therefore used for primary inference (Table 3). After Holm correction across 19 models, food-group differences remained statistically detectable for every variable, and all Kruskal–Wallis sensitivity tests were concordant. The largest effect sizes occurred for carbohydrates, η^2^ = 0.590, 95% CI 0.555–0.632, energy, η^2^ = 0.588, 95% CI 0.530–0.654, total fat, η^2^ = 0.535, 95% CI 0.452–0.626, MUFA, η^2^ = 0.528, 95% CI 0.467–0.609, and protein, η^2^ = 0.504, 95% CI 0.460–0.574.

Among micronutrients, group separation was large for calcium, phosphorus, potassium, sodium, zinc, vitamin C, and folate according to conventional η^2^ thresholds, but bootstrap intervals were wider than those for macronutrients because of missingness and extreme values. Vitamin A had the smallest effect, η^2^ = 0.029, 95% CI 0.016–0.061, despite a significant Welch test after multiplicity correction. This combination indicates statistically detectable group differences with limited explanatory magnitude and replaces the earlier unsupported statement that every ordinary ANOVA was significant without correction.

### 3.4. Robust Patterns of Association Between Nutrients

**Figure 1:**
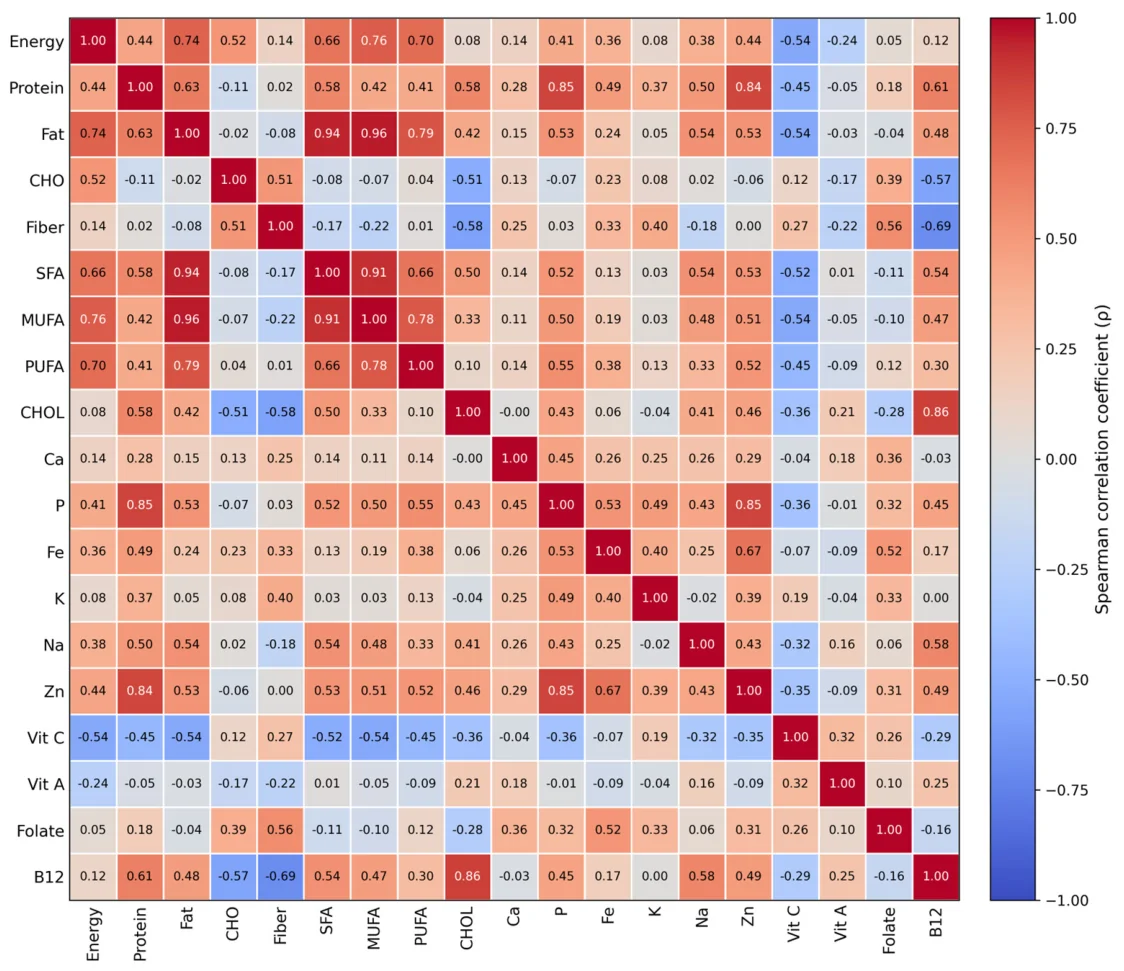
Spearman correlation matrix among the 19 nutritional variables. Note. Cell values are Spearman coefficients. Positive and negative associations are distinguished by the scale. Acronyms are defined in the List of Abbreviations.

The Spearman matrix identified several high-magnitude associations (Figure 1; Table 4). Total fat was strongly associated with MUFA, ρ = 0.956, and SFA, ρ = 0.937, while SFA and MUFA were also strongly correlated, ρ = 0.908. These coefficients primarily reflect the fact that fatty acid fractions are components of total fat and tend to increase together. Energy was associated with total fat, ρ = 0.737, and MUFA, ρ = 0.757, as expected from energy calculation and the high energetic yield of lipids. These relationships were retained for structural completeness but were not interpreted as newly discovered nutrient interactions.

Associations not determined directly by arithmetic were also evident. Protein correlated with phosphorus, ρ = 0.850, and zinc, ρ = 0.836; phosphorus correlated with zinc, ρ = 0.845; and cholesterol correlated with vitamin B12, ρ = 0.862. After adjustment for food group, the corresponding partial rank coefficients were 0.772, 0.746, 0.782, and 0.362, indicating that the mineral–protein relationships persisted more strongly within groups than the cholesterol–vitamin B12 relationship. Fiber and vitamin B12 were inversely correlated, ρ = −0.693, but this association attenuated to −0.133 after controlling for food group, showing that it arose mainly from separation between plant and animal categories. The correlation analysis therefore distinguishes compositional dependence, within-group nutrient co-occurrence, and between-group structure.

### 3.5. Dimensionality Reduction and Nutritional Gradients

**Figure 2:**
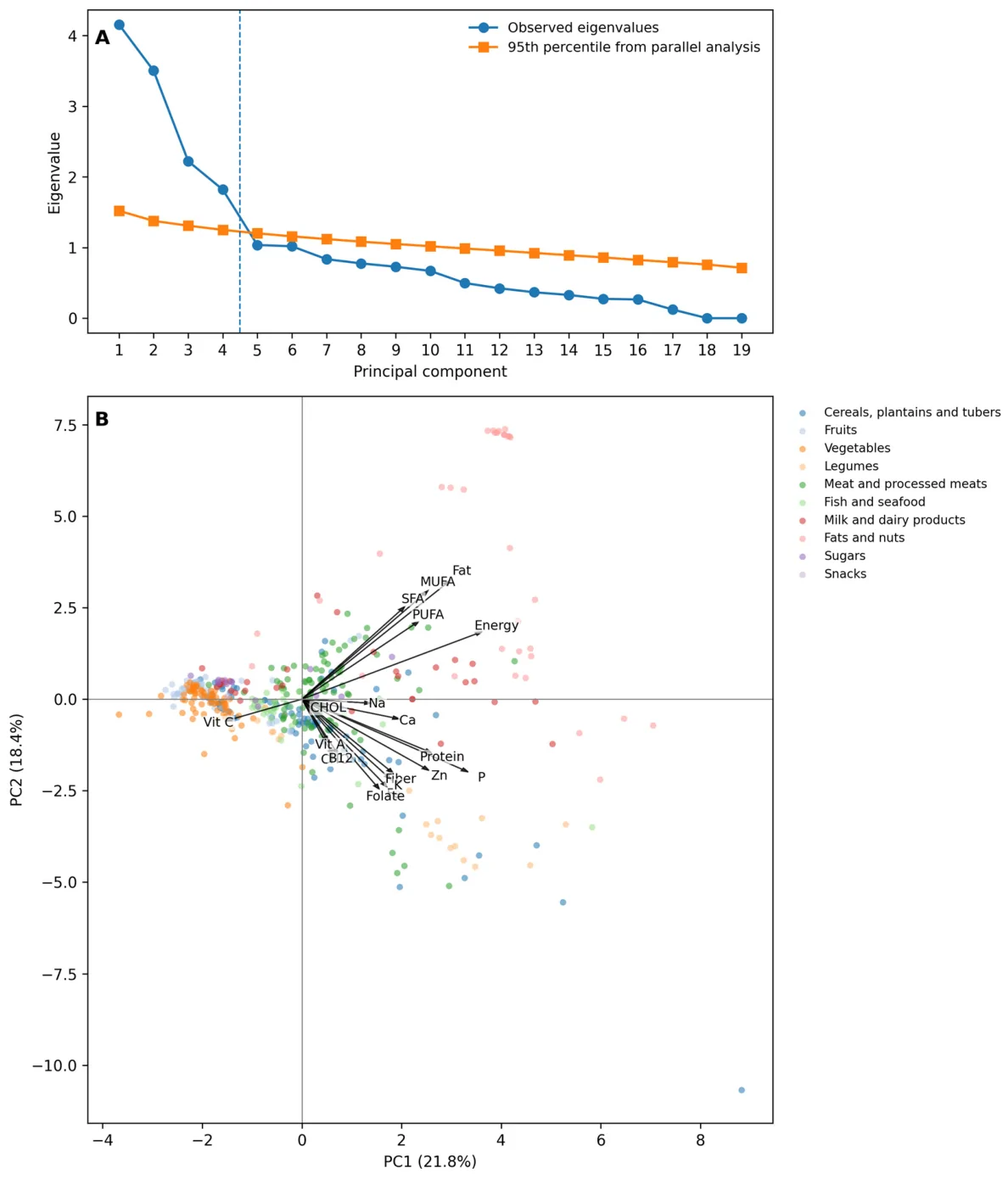
Principal component retention and biplot of the complete-case subset. Note. Panel (**A**) compares observed eigenvalues with the 95th percentile from 1000 parallel-analysis permutations. Panel (**B**) projects 427 complete records and correlation-loading vectors onto PC1 and PC2. The projection represents 40.2% of total variance. The dashed vertical line in panel (**A**) marks the number of components retained by parallel analysis.

Principal component analysis was performed on the 427 complete records. The first two components explained 21.8% and 18.4% of variance, respectively, and the first four explained 61.4%. Horn parallel analysis retained four components because only their eigenvalues exceeded the 95th percentile of permuted reference matrices (Figure 2A). The first five components explained 66.9%, but the fifth was not retained by the parallel criterion. Table 5 reports correlation loadings rather than unscaled eigenvector coefficients.

PC1 had high positive loadings for energy, 0.805, phosphorus, 0.742, total fat, 0.661, protein, 0.579, zinc, 0.567, MUFA, 0.562, and PUFA, 0.522. Vitamin C loaded negatively at −0.344. PC1 is therefore interpreted conservatively as an energy–lipid–mineral covariation axis. It does not establish a general nutrient-density continuum, because nutrient density depends on the nutrient set and reference basis and because PC1 explained only 21.8% of total variance.

PC2 emphasized lipid composition, with positive loadings for total fat, 0.720, MUFA, 0.659, SFA, 0.562, and PUFA, 0.473, opposed by folate, −0.547, iron, −0.534, potassium, −0.487, fiber, −0.450, and phosphorus, −0.442. PC3 contrasted carbohydrates, 0.643, and fiber, 0.586, with vitamin B12, −0.624, vitamin A, −0.540, and cholesterol, −0.537. These axes summarize covariance among variables in the complete-case subset and should not be interpreted as dietary patterns consumed by individuals.

The biplot in Figure 2B shows partial separation of food groups but also substantial overlap. Oils extended toward positive PC1 and PC2 values, while many fruits and vegetables occupied negative PC1 values. Animal-source foods were distributed across positive PC1 and negative PC2 regions. Because the first two components retained only 40.2% of total variance, the projection is descriptive and cannot represent all distances among foods.

### 3.6. Nutritional Typologies Identified Through Clustering

**Figure 3:**
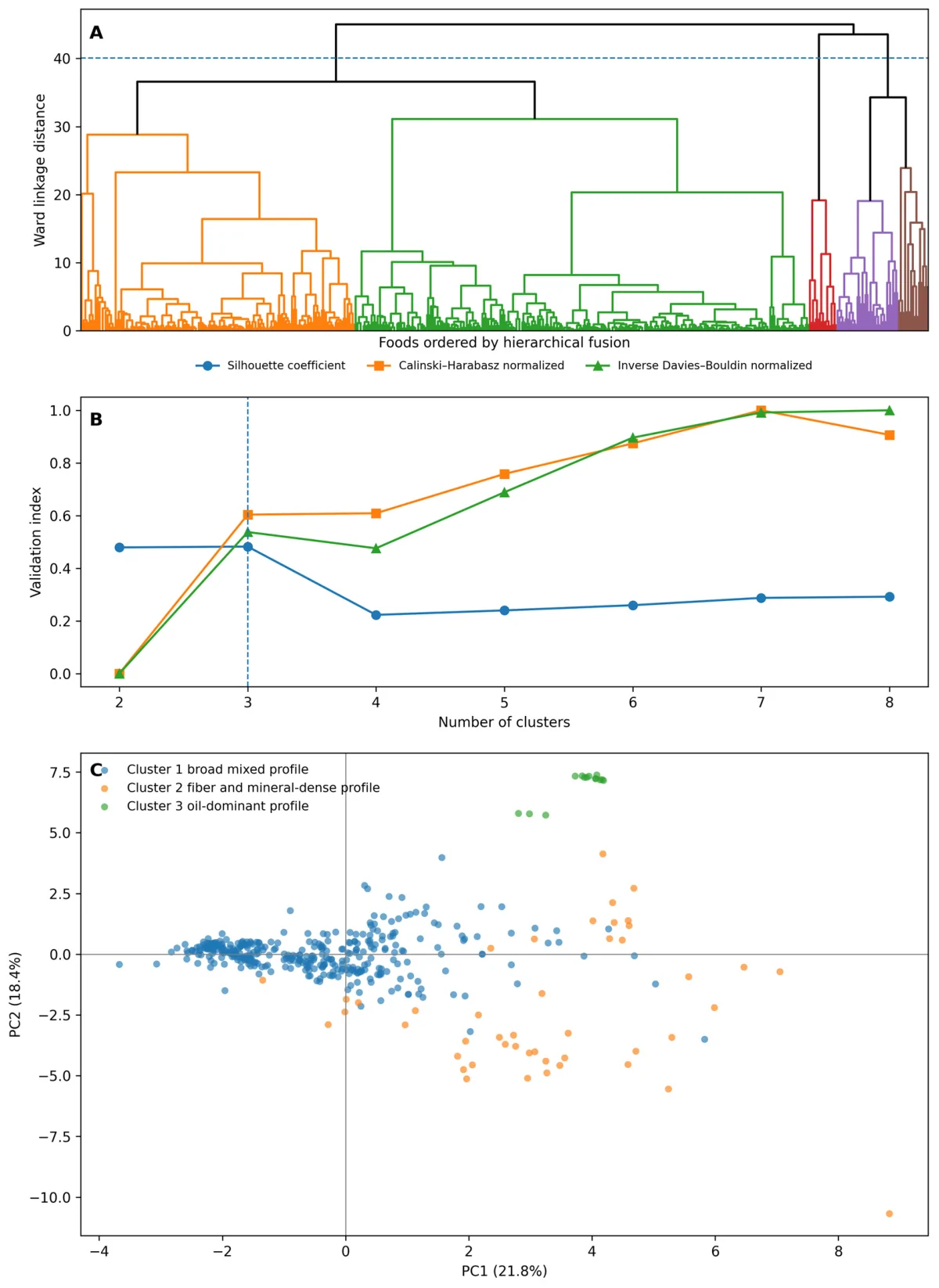
Hierarchical structure, cluster validation, and two-dimensional visualization. Note. Panel (**A**) shows the Ward dendrogram and the cut yielding three clusters; the dashed horizontal line marks the selected cut height, and branch colors are assigned automatically by the clustering algorithm and do not map one-to-one onto the three retained profiles. Panel (**B**) compares full-space validation indices for alternative solutions; the dashed vertical line marks the selected three-cluster solution. Panel (**C**) displays the retained clusters on PC1 and PC2 for interpretation only; validation was performed in the complete 19-dimensional space.

Ward clustering was evaluated in the complete 19-dimensional standardized space. The three-cluster solution had the highest silhouette coefficient among non-degenerate solutions, 0.482, compared with 0.479 for two clusters and 0.223 for four clusters. It also produced a Calinski–Harabasz index of 67.57, a Davies–Bouldin index of 1.63, a minimum cluster size of 14, and median adjusted Rand stability of 0.845, interquartile range 0.758–0.926, across 100 subsamples. On this evidence, three clusters were retained instead of the previously selected four-cluster solution based only on interpretability (Figure 3).

Cluster 1 contained 367 foods and represented a broad mixed profile with generally low-to-moderate standardized nutrient values. It included most meat and processed meats, cereals, vegetables, fruits, dairy products, and fish. Cluster 2 contained 46 foods and showed positive standardized means for potassium, iron, fiber, folate, zinc, and phosphorus. It comprised fats and nuts, legumes, meat products, cereals, vegetables, and fish. Cluster 3 contained 14 oils and was defined by very high total fat, MUFA, PUFA, SFA, and energy, together with negligible protein, carbohydrates, and minerals. Table 6 reports means, standard deviations, and cluster-specific standardized means for all 19 variables used to create the solution.

Figure 3C displays the cluster assignments on PC1 and PC2 only to aid interpretation. Cluster validity was not inferred from this projection. The three-cluster solution was selected from full-space indices and resampling stability, while the dendrogram in Figure 3A documents the hierarchical fusion structure. PCA and Ward clustering used the same standardized variables and are complementary representations of one data matrix, not independent validation methods.

### 3.7. Extreme Cases and Foods of Nutritional Interest

Extreme-value screening identified foods with high recorded concentrations while preserving source provenance (Table 7). Pure vegetable oils occupied the upper energy and total-fat ranges. Dehydrated pork and dried salted beef had the highest protein concentrations, which largely reflect water removal during processing. These observations describe values per 100 g and do not imply superiority at usual serving sizes.

Fennel seed and hard Parmesan cheese had the highest recorded calcium values. Cooked beef blood and the packaged product recorded as “Powder chocolate, Milo” had the highest retained iron values, 61.4 and 60.0 mg/100 g, respectively. The latter should not be renamed cocoa powder because the database identifies a branded malted chocolate-flavored powder. Sweet red pepper and guava had the highest vitamin C values. Fortified breakfast cereals had high folate and zinc values, whereas the highest vitamin B12 values occurred in the record labeled raw ram liver and in cooked beef liver. The narrative retains the database food names and reports the source category for each value.

Extreme values were retained only when they were traceable and passed the revised discontinuity audit. The six implausible micronutrient cells described in Section 2.2 were set to unavailable and do not appear in Table 7.

## 4. Discussion

### 4.1. Food Classification as a Compositional Determinant

The principal finding is not that the USFQ database represents the foods included in the USFQ database, but that its recorded foods exhibit strong compositional organization by food group. Large effects for energy and macronutrients are expected because the categories separate foods with different dominant biochemical matrices, including starch-rich staples, lipid-rich oils, protein-rich animal tissues, and water-rich plant foods [36]. Similar group-level organization was reported in the smaller Cuenca table [10] and in multivariate analyses of national databases elsewhere [7,8]. The consistency across datasets supports the usefulness of food-group labels as a coarse compositional descriptor, while the remaining within-group variance demonstrates that labels cannot replace nutrient-level data.

The expanded nutrient coverage of the USFQ compilation permits evaluation of fatty acid fractions and B-complex vitamins that were unavailable in earlier Ecuadorian tables. This additional dimensionality improves food-level profiling, but it also increases missingness and dependence among variables. The revised complete-case analysis therefore trades breadth of nutrients for reduced representation of beverages, snacks, and sugars [37]. International comparisons should focus on analytical structure and data quality rather than direct differences in mean nutrient content, because databases differ in food definitions, sources, preparation states, fortification practices, and missing-data conventions.

### 4.2. Lipids as the Structuring Axis of Energy Density

The lipid-related correlation structure has a clear phenomenological basis. Total fat is the aggregate mass of fatty acids and other lipid constituents, so positive associations with SFA, MUFA, and PUFA are partly definitional. Energy is also calculated largely from fat, protein, and available carbohydrates, which makes its association with total fat partly arithmetic [38]. The scientific value of these results lies less in their magnitude than in confirming internal coherence and identifying where source heterogeneity or extraction errors would disrupt expected relationships. By contrast, protein–phosphorus and protein–zinc associations persisted after food-group adjustment, suggesting recurrent co-location of these nutrients within biological tissues and food matrices rather than only separation among broad categories.

PC2 separated lipid-rich foods from foods characterized by folate, iron, potassium, fiber, and phosphorus. This pattern is comparable to nutrient-structure analyses reported in other national databases, where ordination commonly distinguishes lipid-dense, protein–mineral, and plant–micronutrient combinations [7,8]. Nevertheless, a principal component is a statistical direction of maximum variance, not a measure of dietary quality. Positive or negative scores do not indicate healthy or unhealthy foods without an explicit nutrient-profiling framework, serving-size basis, and policy objective.

### 4.3. Convergence of Protein and Micronutrients in Animal Sources

The association of protein with phosphorus and zinc is compatible with their co-occurrence in animal tissues, legumes, seeds, and whole grains. The weaker within-group persistence of the cholesterol–vitamin B12 relationship shows that much of that correlation was generated by contrasts between animal and plant categories. These findings illustrate why unadjusted nutrient correlations can be misleading when food groups act as a common structural cause. They also reinforce that compositional co-occurrence does not establish absorption or nutritional equivalence. Iron and zinc bioavailability depends on chemical form, processing, phytate, and meal context [39,40], none of which is represented in the database.

Legumes occupied the fiber- and mineral-dense cluster together with seeds and selected animal products [41,42,43], demonstrating that empirically derived clusters need not reproduce conventional food groups. This cross-group composition is relevant to food education because foods can share nutrient characteristics despite different cultural or biological origins. However, translating this result into dietary guidance requires consumption frequencies, portion sizes, affordability, acceptability, and environmental indicators. The current analysis can identify candidate foods for subsequent assessment, but it cannot quantify their contribution to nutrient adequacy or sustainable diets.

### 4.4. The Footprint of Industrial Fortification on the Nutritional Profile

Fortified breakfast cereals and packaged powders illustrate how industrial formulation can produce micronutrient values that exceed those of unfortified foods. These records are useful for documenting compositional heterogeneity, but product labels may reflect declared values, rounding rules, or fortification targets rather than independent laboratory measurements [44,45]. The study did not assign NOVA categories because ingredient lists and processing information were not systematically available [16]. Consequently, no inference is made about the health effects of ultra-processed foods. The appropriate interpretation is that source and fortification status must accompany nutrient values when databases combine analytical tables and commercial labels.

For public-health practice in Ecuador, the immediate implication is methodological. Harmonized and traceable composition data are prerequisites for estimating intake from dietary surveys, designing food-based guidance, and evaluating reformulation or fortification policies [1,5,46]. The present results can inform which nutrients and food categories require improved analytical coverage and where label-derived values should be distinguished from laboratory measurements. They cannot determine population deficiencies, excess intake, or disease burden because no consumption weights, demographic data, serving sizes, or biomarkers were analyzed.

### 4.5. Complementarity Between Ordination and Multivariate Classification

The three retained clusters summarize the full 19-variable space more parsimoniously than the earlier four-cluster solution. The oil-dominant cluster was highly distinct, while the broad mixed cluster contained foods from several conventional groups [47]. This result shows both the strength and the limitation of unsupervised classification. Ward clustering identifies compact regions according to Euclidean variance, but cluster boundaries depend on scaling, variables, complete-case selection, and the linkage criterion. The validation indices and stability analysis provide quantitative support for three clusters without implying that they are unique natural classes.

From a food-security perspective, compositional diversity is only one component of access to nutritious diets. A database may contain nutrient-dense foods that are geographically unavailable, unaffordable, seasonally constrained, or rarely consumed. The present analysis can support hypothesis generation about foods that merit inclusion in dietary, market, or intervention studies. Evidence concerning food security, nutritional adequacy, or health outcomes must come from designs that integrate composition with consumption, access, socioeconomic conditions, and biological outcomes [48,49,50,51].

### 4.6. Scope and Limitations of the Study

The first limitation is provenance. The database combines nine sources, including foreign tables, USDA records, commercial labels, scientific publications, and recipe calculations. Nutrient values may therefore differ in analytical method, cultivar, geography, preparation, fortification, and date. Record-level traceability improves transparency but does not eliminate source heterogeneity. The results describe values compiled in the USFQ table and should not be generalized to all foods available or consumed in Ecuador.

The second limitation is missingness. Only 427 records contained all 19 variables, and complete-case selection was strongly associated with food group. Beverages were absent and snacks were nearly absent from PCA and clustering, while vegetables and animal-source foods were overrepresented. The observed multivariate gradients and clusters are consequently conditional on the complete-case subset. Multiple imputation was not used because the missing-data mechanism, source-specific measurement processes, and joint distributions could not be justified sufficiently for reliable reconstruction.

The third limitation concerns measurement and model interpretation. Quality-control rules detect gross inconsistencies but cannot verify every upstream analytical value. Means based on very small samples remain unstable even when reported transparently. Welch and rank-based analyses reduce sensitivity to unequal variance and outliers but do not remove dependence created by food-group structure or mathematical relationships among nutrients. PCA and clustering are exploratory, scale-dependent methods, and the first two components represent less than half of total variance. Finally, the database lacks consumption weights, portion sizes, NOVA classification, prices, availability, and health outcomes. Policy applications must therefore be restricted to improving composition-data infrastructure and prioritizing future analytical work.

## 5. Conclusions

This study provides a revised, reproducible characterization of nutritional variation among 996 foods and beverages included in the USFQ Food Chemical Composition Table. Robust group comparisons showed that food-group classification explains substantial variation in energy and major macronutrients, while its explanatory contribution is smaller for vitamin A and more uncertain for sparsely reported micronutrients. Rank correlations separated expected arithmetic dependencies from nutrient co-occurrence that persisted within food groups. Parallel analysis retained four principal components, with the first two summarizing energy–lipid–mineral and lipid-composition covariance but explaining only 40.2% of total variance. Quantitative cluster validation supported three rather than four profiles as follows: a broad mixed profile, a fiber- and mineral-dense profile, and an oil-dominant profile. The analysis also demonstrated that complete-case selection was strongly non-random across food groups, limiting multivariate inference to 427 records and excluding beverages and nearly all snacks. These findings improve the internal documentation and analytical utility of the USFQ database [52]. They can support harmonization, quality control, nutrient profiling, and selection of foods for subsequent research. They do not estimate food availability, dietary intake, nutrient adequacy, processing category, or health effects in Ecuador. Future work should integrate chemically verified Ecuadorian samples with standardized provenance, improved micronutrient completeness, and representative consumption data.

## Figures and Tables

**Table 1:** Distribution of foods by food group.

Food Group	*n*	Percentage (%)
Cereals, plantains and tubers	178	17.9
Fruits	111	11.1
Vegetables	84	8.4
Legumes	36	3.6
Meat and processed meats	123	12.3
Fish and seafood	44	4.4
Milk and dairy products	63	6.3
Fats and nuts	59	5.9
Sugars	89	8.9
Snacks	98	9.8
Beverages and liquids	111	11.1
Total	996	100.0

Note. Values correspond to the 996 records that passed automated quality control and the additional discontinuity filter described in Section 2.2, out of a total of 1002 reconstructed records. Beverages and liquids are reported per 100 mL, and all other groups per 100 g of edible portion.

**Table 2:** Nutritional composition by food group with nutrient-specific sample sizes.

Food Group	Energy (kcal)	Protein (g)	Fat (g)	CHO (g)	Fiber (g)	Calcium (mg)	Iron (mg)	Zinc (mg)
Cereals, plantains and tubers	310.2 ± 123.2 (178)	7.6 ± 4.5 (174)	5.9 ± 7.0 (177)	56.8 ± 21.8 (178)	4.9 ± 5.6 (129)	45.0 ± 60.9 (151)	3.7 ± 5.7 (152)	1.8 ± 2.9 (134)
Fruits	134.6 ± 126.3 (111)	1.5 ± 1.7 (106)	2.3 ± 7.2 (108)	27.3 ± 24.4 (111)	3.8 ± 4.6 (83)	25.7 ± 45.9 (86)	0.7 ± 0.9 (86)	0.2 ± 0.3 (70)
Vegetables	44.9 ± 32.1 (84)	2.3 ± 1.8 (83)	0.3 ± 0.3 (83)	8.3 ± 6.5 (83)	2.6 ± 1.8 (74)	45.8 ± 66.3 (84)	1.7 ± 4.3 (84)	0.4 ± 0.5 (78)
Legumes	263.5 ± 123.2 (36)	18.3 ± 10.2 (36)	3.5 ± 4.7 (36)	39.7 ± 20.6 (36)	13.0 ± 8.7 (29)	105.1 ± 108.2 (34)	5.4 ± 4.0 (34)	2.3 ± 1.3 (30)
Meat and processed meats	208.2 ± 99.2 (123)	20.7 ± 9.0 (123)	13.3 ± 10.7 (123)	1.4 ± 2.8 (123)	0.0 ± 0.2 (101)	24.1 ± 25.1 (120)	3.4 ± 6.8 (119)	2.9 ± 1.5 (102)
Fish and seafood	117.1 ± 43.6 (44)	20.5 ± 7.5 (44)	3.7 ± 3.2 (44)	0.5 ± 1.1 (41)	0.0 ± 0.0 (34)	56.9 ± 90.3 (42)	1.8 ± 2.3 (42)	1.6 ± 2.8 (36)
Milk and dairy products	217.2 ± 142.8 (63)	12.7 ± 9.6 (63)	14.8 ± 12.5 (63)	8.3 ± 11.8 (63)	0.1 ± 0.4 (48)	368.8 ± 320.5 (41)	0.3 ± 0.4 (41)	1.5 ± 1.3 (36)
Fats and nuts	613.0 ± 201.3 (59)	10.5 ± 10.0 (59)	53.4 ± 29.5 (59)	22.5 ± 21.2 (59)	6.6 ± 9.0 (54)	122.4 ± 252.6 (42)	2.9 ± 4.2 (43)	1.9 ± 2.4 (40)
Sugars	268.4 ± 134.6 (89)	1.7 ± 2.7 (86)	3.8 ± 6.3 (87)	57.2 ± 32.9 (89)	0.4 ± 1.1 (47)	54.8 ± 82.8 (24)	4.4 ± 12.9 (24)	0.3 ± 0.5 (16)
Snacks	444.2 ± 143.5 (97)	10.1 ± 13.1 (97)	21.6 ± 11.8 (97)	52.4 ± 24.6 (97)	2.5 ± 4.2 (55)	18.6 ± 11.5 (6)	1.0 ± 1.3 (6)	NR (*n* = 2)
Beverages and liquids	48.8 ± 143.9 (111)	0.2 ± 0.8 (105)	3.0 ± 16.8 (104)	5.6 ± 4.6 (111)	0.2 ± 0.4 (44)	NR (*n* = 1)	NR (*n* = 2)	NA (*n* = 0)

Note. Cells report mean ± standard deviation (*n*) per 100 g of edible portion or per 100 mL for beverages and liquids. NR indicates that a mean was not interpreted because *n* < 3; NA indicates no available observation. CHO = carbohydrates.

**Table 3:** Welch one-way analyses, multiplicity-adjusted significance, and effect sizes for 19 nutritional variables.

Variable	*N*	k	df1	df2	Welch F	pHolm	η^2^ (95% CI)	ω^2^
Energy	995	11	10	310.24	200.46	<0.0001	0.588 (0.530–0.654)	0.583
Protein	976	11	10	291.35	166.29	<0.0001	0.504 (0.460–0.574)	0.498
Total fat	981	11	10	286.09	96.13	<0.0001	0.535 (0.452–0.626)	0.530
Carbohydrates	991	11	10	318.81	226.91	<0.0001	0.590 (0.555–0.632)	0.586
Fiber	698	11	10	219.44	43.60	<0.0001	0.323 (0.264–0.419)	0.313
SFA	836	11	10	242.04	53.74	<0.0001	0.252 (0.206–0.332)	0.243
MUFA	648	11	10	145.30	74.24	<0.0001	0.528 (0.467–0.609)	0.520
PUFA	648	11	10	146.59	31.71	<0.0001	0.379 (0.319–0.479)	0.369
Cholesterol	646	8	7	197.40	29.45	<0.0001	0.163 (0.111–0.425)	0.153
Calcium	630	10	9	102.10	10.64	<0.0001	0.334 (0.242–0.470)	0.324
Phosphorus	594	11	10	25.41	51.48	<0.0001	0.252 (0.215–0.329)	0.239
Iron	633	11	10	29.56	14.60	<0.0001	0.126 (0.067–0.314)	0.112
Potassium	556	10	9	91.54	7.16	<0.0001	0.219 (0.137–0.339)	0.206
Sodium	916	11	10	274.12	29.12	<0.0001	0.189 (0.147–0.295)	0.180
Zinc	544	10	9	29.54	42.64	<0.0001	0.189 (0.145–0.303)	0.175
Vitamin C	571	11	10	24.04	10.34	<0.0001	0.229 (0.194–0.300)	0.215
Vitamin A	540	9	8	147.04	12.51	<0.0001	0.029 (0.016–0.061)	0.015
Folate	463	9	8	124.09	14.76	<0.0001	0.152 (0.102–0.300)	0.137
Vitamin B12	423	8	7	97.58	12.72	<0.0001	0.100 (0.078–0.153)	0.085

Note. *N* is the total nutrient-specific sample size; k is the number of groups included in the Welch model; df1 and df2 are Welch degrees of freedom; pHolm is adjusted across 19 tests; η^2^ is reported with a stratified-bootstrap 95% confidence interval; ω^2^ is the less biased descriptive effect-size estimator. Groups with *n* < 2 or zero variance were omitted from the nutrient-specific Welch model.

**Table 4:** Strongest Spearman nutrient correlations ranked by absolute ρ.

Variable 1	Variable 2	ρ	Pearson r	Partial ρ	pHolm	*n*
Total fat	MUFA	0.956	0.882	0.894	<0.0001	648
Total fat	SFA	0.937	0.702	0.889	<0.0001	836
SFA	MUFA	0.908	0.510	0.831	<0.0001	648
Cholesterol	Vitamin B12	0.862	0.285	0.362	<0.0001	475
Protein	Phosphorus	0.850	0.662	0.772	<0.0001	582
Phosphorus	Zinc	0.845	0.527	0.782	<0.0001	529
Protein	Zinc	0.836	0.493	0.746	<0.0001	533
Total fat	PUFA	0.787	0.732	0.687	<0.0001	648
MUFA	PUFA	0.782	0.534	0.666	<0.0001	647
Energy	MUFA	0.757	0.736	0.625	<0.0001	648
Energy	Total fat	0.737	0.790	0.616	<0.0001	981
Energy	PUFA	0.701	0.610	0.527	<0.0001	648
Fiber	Vitamin B12	−0.693	−0.095	−0.133	<0.0001	460
Iron	Zinc	0.674	0.469	0.654	<0.0001	541
SFA	PUFA	0.665	0.273	0.566	<0.0001	648
Energy	SFA	0.661	0.564	0.553	<0.0001	836
Protein	Total fat	0.632	0.194	0.438	<0.0001	975
Protein	Vitamin B12	0.608	0.226	0.067	<0.0001	484
Protein	SFA	0.585	0.170	0.367	<0.0001	835
Sodium	Vitamin B12	0.579	0.044	0.320	<0.0001	484

Note. pHolm is adjusted across 171 pairwise Spearman tests. Pearson r is shown as a sensitivity estimate. Partial ρ was calculated after residualizing nutrient ranks for food-group indicators. Sample size varies because pairwise-available observations were used.

**Table 5:** Correlation loadings of the first three principal components.

Variable	PC1	PC2	PC3
Energy	0.805	0.410	0.275
Protein	0.579	−0.325	−0.393
Total fat	0.661	0.720	−0.004
Carbohydrates	0.138	−0.339	0.643
Dietary fiber	0.408	−0.450	0.586
SFA	0.459	0.562	−0.168
MUFA	0.562	0.659	0.014
PUFA	0.522	0.473	0.179
Cholesterol	0.109	−0.050	−0.537
Calcium	0.437	−0.120	−0.094
Phosphorus	0.742	−0.442	−0.109
Iron	0.380	−0.534	0.057
Potassium	0.397	−0.487	0.237
Sodium	0.311	−0.025	−0.265
Zinc	0.567	−0.433	−0.134
Vitamin C	−0.344	−0.134	0.058
Vitamin A	0.117	−0.257	−0.540
Folate	0.346	−0.547	0.184
Vitamin B12	0.161	−0.335	−0.624

Note. Loadings are correlations between standardized nutrient variables and component scores. PC1, PC2, and PC3 explained 21.8%, 18.4%, and 11.7% of total variance, respectively. Horn parallel analysis retained four components.

**Table 6:** Profiles of the three Ward clusters across all 19 variables.

Variable	Cluster 1 (*n* = 367)	Cluster 2 (*n* = 46)	Cluster 3 (*n* = 14)
Energy (kcal/100 g or mL)	179.93 ± 134.01 (−0.23)	381.43 ± 201.29 (+0.81)	863.83 ± 70.92 (+3.30)
Protein (g/100 g or mL)	10.01 ± 9.39 (−0.07)	19.66 ± 8.62 (+0.91)	0.19 ± 0.37 (−1.08)
Total fat (g/100 g or mL)	6.60 ± 9.74 (−0.21)	18.99 ± 22.39 (+0.41)	95.87 ± 8.11 (+4.27)
Carbohydrates (g/100 g or mL)	20.11 ± 27.20 (−0.03)	32.96 ± 25.24 (+0.45)	0.07 ± 0.24 (−0.77)
Dietary fiber (g/100 g or mL)	2.10 ± 2.97 (−0.17)	11.13 ± 10.45 (+1.56)	0.00 ± 0.00 (−0.58)
SFA (g/100 g or mL)	2.61 ± 4.98 (−0.11)	2.70 ± 2.99 (−0.10)	27.41 ± 24.08 (+3.11)
MUFA (g/100 g or mL)	2.48 ± 3.97 (−0.19)	8.01 ± 13.18 (+0.41)	37.90 ± 19.93 (+3.65)
PUFA (g/100 g or mL)	0.94 ± 1.75 (−0.21)	6.93 ± 10.15 (+0.64)	26.25 ± 21.72 (+3.35)
Cholesterol (mg/100 g or mL)	49.77 ± 189.88 (−0.00)	60.04 ± 137.93 (+0.05)	37.50 ± 79.32 (−0.07)
Calcium (mg/100 g or mL)	66.08 ± 141.01 (−0.03)	124.72 ± 176.66 (+0.38)	5.64 ± 11.12 (−0.45)
Phosphorus (mg/100 g or mL)	139.80 ± 136.33 (−0.14)	413.43 ± 261.52 (+1.41)	5.07 ± 10.08 (−0.90)
Iron (mg/100 g or mL)	1.59 ± 1.75 (−0.19)	8.54 ± 7.99 (+1.66)	0.05 ± 0.15 (−0.59)
Potassium (mg/100 g or mL)	246.35 ± 163.34 (−0.17)	780.25 ± 545.11 (+1.67)	6.50 ± 13.37 (−1.00)
Sodium (mg/100 g or mL)	222.57 ± 513.71 (+0.03)	130.63 ± 208.40 (−0.16)	109.43 ± 284.67 (−0.21)
Zinc (mg/100 g or mL)	1.30 ± 1.38 (−0.15)	4.53 ± 3.87 (+1.41)	0.02 ± 0.04 (−0.76)
Vitamin C (mg/100 g or mL)	8.71 ± 20.32 (+0.02)	7.85 ± 13.56 (−0.03)	0.00 ± 0.00 (−0.43)
Vitamin A (µg RAE/100 g or mL)	51.87 ± 130.43 (−0.13)	1050.20 ± 2468.58 (+1.02)	156.21 ± 311.92 (−0.01)
Folate (µg/100 g or mL)	37.91 ± 67.57 (−0.16)	287.86 ± 379.18 (+1.41)	0.50 ± 1.09 (−0.40)
Vitamin B12 (µg/100 g or mL)	1.05 ± 2.91 (−0.11)	9.66 ± 20.97 (+0.99)	0.03 ± 0.06 (−0.25)

Note. Cells report mean ± standard deviation with the cluster-specific standardized mean in parentheses. Clusters were derived from all 19 Z-standardized variables. Cluster sizes were 367, 46, and 14.

**Table 7:** Highest retained nutrient values with record-level source provenance.

Nutrient	Food	Food Group	Value	Source
Energy (kcal/100 g or mL)	Oil, avocado	Fats and nuts	900.00	[21]
Energy (kcal/100 g or mL)	Oil, sesame	Fats and nuts	900.00	[17]
Protein (g/100 g or mL)	Pork meet, dehydrated, spicy, smoked, Ferbola	Meat and processed meats	78.57	Nutrition label of packaged product
Protein (g/100 g or mL)	Beef, dried meat, salty	Meat and processed meats	64.80	[17]
Total fat (g/100 g or mL)	Oil, avocado	Fats and nuts	100.00	[21]
Total fat (g/100 g or mL)	Oil, sesame	Fats and nuts	100.00	[17]
Dietary fiber (g/100 g or mL)	Wheat, wheat bran, raw	Cereals, plantains and tubers	42.80	[21]
Dietary fiber (g/100 g or mL)	Fennel seed	Fats and nuts	39.80	[21]
Calcium (mg/100 g or mL)	Fennel seed	Fats and nuts	1196.00	[21]
Calcium (mg/100 g or mL)	Cheese, parmesan, hard	Milk and dairy products	1184.00	[17]
Iron (mg/100 g or mL)	Beef, blood, cooked	Meat and processed meats	61.40	[20]
Iron (mg/100 g or mL)	Powder chocolate, Milo	Sugars	60.00	Nutrition label of packaged product
Zinc (mg/100 g or mL)	Cereal, breakfast, froot loops, trix, Kellogg’s	Cereals, plantains and tubers	19.00	[17]
Zinc (mg/100 g or mL)	Cereal, breakfast, crispy rice	Cereals, plantains and tubers	18.29	[21]
Vitamin C (mg/100 g or mL)	Peppers, red, sweet, fresh, raw	Vegetables	190.00	[17]
Vitamin C (mg/100 g or mL)	Guavas, ordinary, ripe, raw	Fruits	183.00	[17]
Folate (µg/100 g or mL)	Cereal, breakfast, wheat flakes, All-bran	Cereals, plantains and tubers	2198.00	[17]
Folate (µg/100 g or mL)	Cereal, breakfast, Special K, Kellogg’s	Cereals, plantains and tubers	1290.00	[21]
Vitamin B12 (µg/100 g or mL)	Ram, liver, raw	Meat and processed meats	90.05	[17]
Vitamin B12 (µg/100 g or mL)	Beef, liver, cooked	Meat and processed meats	70.58	[17]

Note. Values are reported per 100 g of edible portion or per 100 mL for beverages. Names reproduce the English food labels in the reconstructed database. Six discontinuous micronutrient cells were recorded as unavailable before ranking. Bracketed numbers identify the published food composition table used as source, corresponding to the numbered reference list (Central American table [17]; Bolivian table [20]; USDA FoodData Central [21]); entries reading “Nutrition label of packaged product” instead indicate a value transcribed directly from a commercial product’s label rather than from a published table, and are not assigned a reference number.

## Data Availability

The original contributions presented in this study are included in the article/Supplementary Materials. Further inquiries can be directed to the corresponding author.

## Supplementary Materials

The following supporting information can be downloaded at https://www.mdpi.com/article/10.3390/nu18172910/s1, Supplementary File S1 contains the reconstructed and audited database and completeness tables. Supplementary File S2 contains executable statistical analysis code and package specifications.

## Abbreviations

The following abbreviations are used in the text, tables, and figures of this article.

**Abbreviation**	**Meaning**
CHO	Total carbohydrates
SFAs	Saturated fatty acids
MUFAs	Monounsaturated fatty acids
PUFAs	Polyunsaturated fatty acids
CHOL	Cholesterol
Ca	Calcium
P	Phosphorus
Fe	Iron
K	Potassium
Na	Sodium
Zn	Zinc
Vit C	Vitamin C
Vit A	Vitamin A in retinol activity equivalents
B12	Vitamin B12
PCA	Principal component analysis
PC1, PC2, PC3	First, second, and third principal components
ANOVA	Analysis of variance
η^2^	Eta-squared effect size
ω^2^	Omega-squared effect size
USFQ	Universidad San Francisco de Quito
QC	Quality control
